# Jordan–Wigner transformations for tree structures

**DOI:** 10.1038/s41598-018-38128-8

**Published:** 2019-02-22

**Authors:** Stefan Backens, Alexander Shnirman, Yuriy Makhlin

**Affiliations:** 1 Institut für Theorie der Kondensierten Materie, Karlsruhe Institute of Technology, D-76131 Karlsruhe, Germany; 2 Institute of Nanotechnology, Karlsruhe Institute of Technology, D-76344 Eggenstein-Leopoldshafen, Germany; 3 Condensed-matter physics Laboratory, National Research University Higher School of Economics, 101000 Moscow, Russia; 4 Landau Institute for Theoretical Physics, acad. Semyonov av. 1a, 142432 Chernogolovka, Russia

## Abstract

The celebrated Jordan–Wigner transformation provides an efficient mapping between spin chains and fermionic systems in one dimension. Here we extend this spin–fermion mapping to arbitrary *tree* structures, which enables mapping between fermionic and spin systems with nearest-neighbor coupling. The mapping is achieved with the help of additional spins at the junctions between one-dimensional chains. This property allows for straightforward simulation of Majorana braiding in spin or qubit systems.

## Introduction

The well-known Jordan–Wigner transformation relates spin-$\frac{1}{2}$ operators to fermionic creation and annihilation operators. Thereby, it allows for mapping between spin and fermionic systems. It was originally used by Jordan and Wigner to define fermionic operators in the second quantization^1^. The Jordan–Wigner transformation introduces non-local “string operators” to transform commuting operators of different spins into anti-commuting fermionic operators, and, in general, does not preserve locality. Nevertheless, it maps even-parity local fermionic Hamiltonians to local spin Hamiltonians; moreover, certain spin Hamiltonians in 1D (one dimension) are mapped to free fermionic Hamiltonians, which are readily solvable^2^.

Generalizations to higher dimensions were discussed in recent decades^3–8^. They map (even-parity) fermionic Hamiltonians to spin Hamiltonians, but even local quadratic fermionic terms are mapped onto operators which involve many, in principle infinitely many, spin operators (though in some cases^3,5^ the weight of the involved spins may slowly decay with distance). One may also consider introducing ancillary degrees of freedom. For instance, Verstrate and Cirac^7^ suggested doubling the number of degrees of freedom for a 2D lattice to achieve local, but not necessarily simple Hamiltonians in the spin language.

Thus, free fermionic Hamiltonians are often mapped to complicated operators in the spin language. In ref.^9^, a modified Jordan–Wigner transformation was proposed, such that a three-leg star graph of free fermions (with nearest-neighbor hopping) could be mapped to a three-leg star graph of spin chains (with nearest-neighbor couplings). The mapping required the introduction of an extra spin-$\frac{1}{2}$ in the vertex of the spin graph, coupled to the three spin chains locally via a specific 3-spin coupling. Furthermore, in ref.^9^ an alternative scenario was described, in which a three-leg spin graph with exclusively 2-spin interactions was mapped to a Kondo-like system of fermionic chains coupled by one spin (cf. application in refs^10,11^). Here, we demonstrate that these transformations can be generalised to binary-tree structures of 1D chains, connected, acyclic graphs with no more than three edges at each vertex. Furthermore, we argue that this result can be directly generalized to generic, non-binary trees.

This kind of transformation is of special interest in particular since it can be used to simulate the physics and, notably, non-abelian statistics and braiding of fermionic Majorana modes^12,13^ in a (topologically non-protected) spin system. For the case of a *T*-junction geometry with a single topological segment in the chain providing two Majorana modes, this implementation was explicated in ref.^14^. Here we describe braiding operations between Majorana modes belonging to different topological segments in a system where the number of segments is arbitrary. A binary-tree structure may be viewed as consisting of many *T*-junctions; such structures may be useful for implementation (physical simulation) of the Majorana braiding operation^15,16^ with applications in topological quantum computing. We also argue that such spin systems mimic fermionic quantum computers^17^, which can be efficient, e.g., in quantum-chemistry simulations. Namely, braiding or other logic gates between remote qubits naturally include Jordan–Wigner string operators, making these qubits fermionic. Encoding the population of molecular orbitals in such qubits (see e.g., refs^18,19^) thus brings a considerable advantage for the computing algorithms.

An explicit description of a Majorana braiding operation between two topological segments, implemented in the corresponding spin system, is given in the section Methods.

## Results

### Geometry and notations

**Figure 1:**
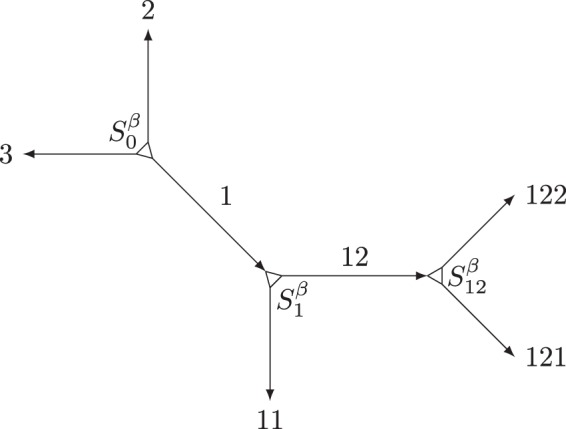
Binary-tree structure consisting of fermionic or spin chains. In each chain, spins or fermions are numbered in the direction of the arrow. The descendent chains in the tree are identified by sequential numbers that are appended to the label of their parent chain. The root is the only vertex with no incoming edge and may have three outgoing edges. The notations for the vertices and the chains are explained in the text and evident from the diagram.

We consider spins on a tree-like lattice of the type depicted in Fig. 1. Each edge of the tree is a one-dimensional spin chain. The chains are connected at the vertices, and the whole structure indicates the notion of locality (in fact, we focus on nearest-neighbor couplings). In binary trees, they are connected in triples and, in general, interactions between boundary spins from all three chains are allowed, so-called Δ-junctions^10^, indicating all three pairwise couplings. In the particular case when one of the three couplings in the junction vanishes, we obtain a *T*-junction, where all three chains have a common boundary spin. We also consider fermions on the same tree and discuss methods to convert between spin and fermionic systems.

A priori, the tree structures do not have a distinctive root and the edges do not have orientation. For the purposes of the transformation, however, we choose an arbitrary vertex as a root and assign to each edge (chain) an orientation away from the root. Based on this hierarchy, we introduce a notation for our further discussion by assigning a name to each vertex and chain in the tree: The root is denoted “0” and the three outgoing chains acquire numbers 1, 2, and 3. Then, step by step, each other vertex acquires a name *α*, identical with the incoming chain, while the two outgoing chains are assigned a longer name, *αβ*, with *β* = 1 or 2, see Fig. 1.

According to the orientation, the spins or fermions in each chain *α* are numbered from 1 to its length *L*_*α*_; they are represented by the Pauli matrices ${\sigma }_{\alpha }^{x,y,z}(j)$ and the fermionic creation/annihilation operators ${c}_{\alpha }^{\dagger }(j)/{c}_{\alpha }(j)$, respectively. To construct a fermion–spin transformation, we shall need ancillary spin operators, one per chain, which we assign to the vertex at the beginning of the chain. The corresponding Pauli matrices ${S}_{\alpha }^{\beta }$ are labelled with the vertex index *α* and the chain number 1, 2, or 3. An example is depicted in Fig. 1. The spin operators ${S}_{\alpha }^{\mathrm{1,2,3}}$ at each vertex *α* are spin components of the ancillary spin at this vertex.

To describe a fermion-spin transformation, we use separate Jordan–Wigner transformations for each chain *α*,

1a $${c}_{\alpha }(j)={\eta }_{\alpha }[\prod _{k=1}^{j-1}{\sigma }_{\alpha }^{z}(j)]{\sigma }_{\alpha }^{-}(j)$$

1b $${c}_{\alpha }^{\dagger }(j)={\eta }_{\alpha }[\prod _{k=1}^{j-1}{\sigma }_{\alpha }^{z}(k)]{\sigma }_{\alpha }^{+}(j),$$

where ${\sigma }_{\alpha }^{\pm }(j)=\frac{1}{2}[{\sigma }_{\alpha }^{x}(j)\pm i{\sigma }_{\alpha }^{y}(j)]$. The Klein factors *η*_*α*_, with ${\eta }_{\alpha }^{2}=1$, are to be chosen to ensure proper (anti-)commutation relations between operators in different chains^20,21^; they are discussed later. Similar to the standard Jordan–Wigner transformation, these relations ensure that a local quadratic fermionic Hamiltonian is also a local operator in the spin language (this does not hold if cycles are present). In particular, a useful corollary of these definitions,

2 $${\sigma }_{\alpha }^{z}(j)=2{c}_{\alpha }^{\dagger }(j){c}_{\alpha }(j)-1=1-2{c}_{\alpha }(j){c}_{\alpha }^{\dagger }(j)\,,$$

shows that a (magnetic) field in *z*-direction corresponds to a local chemical potential at a fermionic site.

### Free fermions and 3-spin couplings

To complete the description of the transformation, we need to define the operators *η*_*α*_. For the chains directly at the root, *β* = 1, 2, 3, we define the transformation exactly like in ref.^9^:

3a $${\eta }_{\beta }={S}_{0}^{\beta }\mathrm{.}$$

For any other chain, denoted by *αβ* with the parent chain α and *β* = 1, 2, the following definition applies:

3b $${\eta }_{\alpha \beta }={\eta }_{\alpha }[\prod _{k=1}^{{L}_{\alpha }}{\sigma }_{\alpha }^{z}(k)]{S}_{\alpha }^{\beta }\mathrm{\ .}$$

These definitions satisfy the conditions stated in the previous section.

Let us now consider various nearest-neighbour quadratic fermionic couplings and their spin counterparts under the constructed transformation. Within any one-dimensional chain, the Jordan–Wigner transformation is known to convert local quadratic fermionic Hamiltonians into local quadratic spin Hamiltonians; the factors ${\eta }_{\alpha }^{2}=1$ in eq. (1) do not affect this. Therefore we will examine only the couplings at the vertices between different chains. There are two kinds of vertex couplings: those between a parent and a descendant chain and those between two descendant chains of the same parent. A coupling term of the first kind between chains *α* and *αβ* (with *β* = 1, 2) has the general form

4a $${H}_{\alpha ,\alpha \beta }=u{c}_{\alpha }({L}_{\alpha }){c}_{\alpha \beta }\mathrm{(1)}+t{c}_{\alpha }^{\dagger }({L}_{\alpha }){c}_{\alpha \beta }\mathrm{(1)}+{\rm{h}}.{\rm{c}}.,$$

which is transformed, using the relation (1), into

4b $${H}_{\alpha ,\alpha \beta }^{{\rm{S}}}={S}_{\alpha }^{\beta }[u{\sigma }_{\alpha }^{-}({L}_{\alpha }){\sigma }_{\alpha \beta }^{-}\mathrm{(1)}-t{\sigma }_{\alpha }^{+}({L}_{\alpha }){\sigma }_{\alpha \beta }^{-}\mathrm{(1)}+{\rm{h}}.{\rm{c}}].$$

A coupling of the second kind between chains *αβ* and *αγ* (here *β* ≠ *γ*; *β* and *γ* can be 1 or 2; at the root, *α* is empty and $\beta ,\,\gamma =\mathrm{1,}\,\mathrm{2,}\,3$ with ancillary spin operators ${S}_{0}^{\beta }$) has the general form

5a $${H}_{\alpha \beta ,\alpha \gamma }=u{c}_{\alpha \beta }\mathrm{(1)}{c}_{\alpha \gamma }\mathrm{(1)}+t{c}_{\alpha \beta }^{\dagger }\mathrm{(1)}{c}_{\alpha \gamma }\mathrm{(1)}+{\rm{h}}.{\rm{c}}.,$$

which is similarly mapped to

5b $$\begin{array}{ccc}{H}_{\alpha \beta ,\alpha \gamma }^{{\rm{S}}} & = & {S}_{\alpha }^{\beta }\,{S}_{\alpha }^{\gamma }[u{\sigma }_{\alpha \beta }^{-}(1){\sigma }_{\alpha \gamma }^{-}(1)+t{\sigma }_{\alpha \beta }^{+}(1){\sigma }_{\alpha \gamma }^{-}(1)]+{\rm{h}}.\,{\rm{c}}.\\  & = & {S}_{\alpha }^{\nu }{\varepsilon }_{\beta \gamma \nu }[{\rm{i}}u{\sigma }_{\alpha \beta }^{-}(1){\sigma }_{\alpha \gamma }^{-}(1)+{\rm{i}}\,t{\sigma }_{\alpha \beta }^{+}(1){\sigma }_{\alpha \gamma }^{-}(1)+{\rm{h}}.\,{\rm{c}}.\,],\end{array}$$

where ${\varepsilon }_{\beta \gamma \nu }$ is the Levi-Civita symbol.

Let us note that the transformation described can be generalized to arbitrary tree structures, beyond binary trees. Indeed, any higher-order vertex (with more that three edges) can be thought of as built out of three-edge vertices. For instance, Fig. 1 can be viewed as a five-edge vertex, which allows us to define the Klein factors for all chains outside of this figure: In that case, the internal chains in Fig. 1 are of length zero and do not contribute products to the Klein factors, but coupling terms involving more than three spins may appear.

### XY spin system and fermionic Kondo model

In this section, we consider a tree structure of spins with local XY couplings and use the Jordan–Wigner transformation backwards in order to find the corresponding fermionic problems. For a single 1D chain, the Jordan–Wigner transformation maps these to free fermions. In order to find the corresponding fermionic Hamiltonian for a tree structure, we use the generalized Jordan–Wigner transformation defined in eqs (1) and (3). These involve ancillary spin operators ${S}_{\alpha }^{\beta }$, which commute with local spins *σ*(*j*), but not with the fermions *c*(*j*). We show below that the original XY spin model is equivalent to a Kondo-type model on the same tree with one impurity spin per vertex.

To simplify the resulting fermionic Hamiltonians, we introduce, instead of *S*, other spin operators at the inner vertices, ${\tilde{S}}_{\alpha }^{\beta }$. We define

6 $${S}_{0}^{\beta }={\mathop{S}\limits^{ \sim }}_{0}^{\beta }\prod _{{\scriptstyle \begin{array}{c}{\rm{c}}{\rm{h}}{\rm{a}}{\rm{i}}{\rm{n}}\,{\rm{l}}{\rm{a}}{\rm{b}}{\rm{e}}{\rm{l}}{\rm{s}}\,\gamma \\ {\rm{n}}{\rm{o}}{\rm{t}}\,{\rm{b}}{\rm{e}}{\rm{g}}{\rm{i}}{\rm{n}}{\rm{n}}{\rm{i}}{\rm{n}}{\rm{g}}\\ {\rm{w}}{\rm{i}}{\rm{t}}{\rm{h}}\,\beta \end{array}}}{P}_{\gamma }\,\,{\rm{a}}{\rm{n}}{\rm{d}}\,\,{S}_{\alpha }^{\beta }={\mathop{S}\limits^{ \sim }}_{\alpha }^{\beta }\prod _{{\scriptstyle \begin{array}{c}{\rm{c}}{\rm{h}}{\rm{a}}{\rm{i}}{\rm{n}}\,{\rm{l}}{\rm{a}}{\rm{b}}{\rm{e}}{\rm{l}}{\rm{s}}\,\alpha \gamma \\ {\rm{n}}{\rm{o}}{\rm{t}}\,{\rm{b}}{\rm{e}}{\rm{g}}{\rm{i}}{\rm{n}}{\rm{n}}{\rm{i}}{\rm{n}}{\rm{g}}\\ {\rm{w}}{\rm{i}}{\rm{t}}{\rm{h}}\,\alpha \beta \end{array}}}{P}_{\alpha \gamma },$$

where we introduced the notation

7 $${P}_{\alpha }=\prod _{k=1}^{{L}_{\alpha }}{\sigma }_{\alpha }^{z}(k)$$

for the fermionic parity of chain *α*. As the products consist of Pauli matrices *σ*^*z*^ only, operators ${S}_{\alpha }^{\beta }$ inherit the commutation relations of ${\tilde{S}}_{\alpha }^{\beta }$. In other words, $\tilde{S}$ are spin-1/2 operators, and one can verify that they commute with the fermionic operators.

Let us illustrate this with the example of Fig. 1:

8a $${S}_{0}^{1}={\tilde{S}}_{0}^{1}{P}_{2}{P}_{3}$$

8b $${S}_{0}^{2}={\tilde{S}}_{0}^{2}[{P}_{1}{P}_{11}({P}_{12}{P}_{121}{P}_{122})]{P}_{3}$$

8c $${S}_{0}^{3}={\tilde{S}}_{0}^{3}[{P}_{1}{P}_{11}({P}_{12}{P}_{121}{P}_{122})]{P}_{2}$$

(the grouping highlights the tree structure),

9a $${S}_{1}^{1}={\tilde{S}}_{1}^{1}({P}_{12}{P}_{121}{P}_{122})$$

9b $${S}_{1}^{2}={\tilde{S}}_{1}^{2}{P}_{11}$$

9c $${S}_{1}^{3}={\tilde{S}}_{1}^{3}{P}_{11}({P}_{12}{P}_{121}{P}_{122})$$

and

10a $${S}_{1}^{1}={\tilde{S}}_{12}^{1}{P}_{122}$$

10b $${S}_{1}^{2}={\tilde{S}}_{12}^{2}{P}_{121}$$

10c $${S}_{1}^{3}={\tilde{S}}_{12}^{3}{P}_{121}{P}_{122}\mathrm{.}$$

The string (parity) operators guarantee that ${S}_{\alpha }^{\beta }$ commute with the fermionic operators of all chains.

Again, the Jordan–Wigner transformation is known to map XY-coupled spins in a 1D chain to free fermions, so we only have to examine the two kinds of vertex couplings, as we did in the preceding section. They result in Kondo-like couplings of the fermionic chains:

11a $${H}_{\alpha ,\alpha \beta }=u{\sigma }_{\alpha }^{-}({L}_{\alpha }){\sigma }_{\alpha \beta }^{-}\mathrm{(1)}+t{\sigma }_{\alpha }^{+}({L}_{\alpha })\,{\sigma }_{\alpha \beta }^{-}\mathrm{(1)}+{\rm{h}}.{\rm{c}}.$$

11b $$\to {H}_{\alpha ,\alpha \beta }^{{\rm{F}}}={S}_{\alpha }^{\beta }[u{c}_{\alpha }({L}_{\alpha }){c}_{\alpha \beta }\mathrm{(1)}-t{c}_{\alpha }^{\dagger }({L}_{\alpha }){c}_{\alpha \beta }\mathrm{(1)}+{\rm{h}}.{\rm{c}}.]$$

and

12a $${H}_{\alpha \beta ,\alpha \gamma }=u{\sigma }_{\alpha \beta }^{-}\mathrm{(1)}{\sigma }_{\alpha \gamma }^{-}\mathrm{(1)}+t{\sigma }_{\alpha \beta }^{+}\mathrm{(1)}{\sigma }_{\alpha \gamma }^{-}\mathrm{(1)}+{\rm{h}}.{\rm{c}}.$$

12b $$\begin{array}{rcl}\to {H}_{\alpha \beta ,\alpha \gamma }^{{\rm{F}}} & = & {S}_{\alpha }^{\beta }{S}_{\alpha }^{\gamma }[u{c}_{\alpha \beta }\mathrm{(1)}\,{c}_{\alpha \gamma }\mathrm{(1)}+t{c}_{\alpha \beta }^{\dagger }\mathrm{(1)}{c}_{\alpha \gamma }\mathrm{(1)}]+{\rm{h}}.{\rm{c}}.\\  & = & {S}_{\alpha }^{\nu }{\varepsilon }_{\beta \gamma \nu }\,[{\rm{i}}u{c}_{\alpha \beta }\mathrm{(1)}{c}_{\alpha \gamma }\mathrm{(1)}+{\rm{i}}\,t{c}_{\alpha \beta }^{\dagger }\mathrm{(1)}{c}_{\alpha \gamma }\mathrm{(1)}+{\rm{h}}.{\rm{c}}]\mathrm{\ .}\end{array}$$

Thus, inter-chain couplings are controlled by the ancillary spins.

### Majorana braiding and the spin representation

In this section, we are interested in spin implementation of free-fermion models on tree structures. These can be applied, in particular, to realize (physically simulate) Majorana qubits and quantum logical operations using ordinary quantum bits.

Majorana modes arising in the topological phase of the Kitaev chain^22^, a one-dimensional fermionic system, can be braided in a *T*-junction geometry by local tuning of the chemical potential^15^. One can see from the discussion above that similar to refs^9,14^, the corresponding spin model involves Ising couplings within the chains, the ancillary-spin-controlled Ising couplings at the junctions as well as a transverse magnetic field.

In the following, the spin indices are swapped for convenience, to ensure the resulting *zz* Ising couplings and the transverse field in the *x* direction. Furthermore, we use fermionic Majorana operators *γ*_*α*_(*m*), which are connected to the usual fermionic creation and annihilation operators *c*^†^, *c* in the following way: ${\gamma }_{\alpha }\mathrm{(2}j-\mathrm{1)}={c}_{\alpha }(j)+{c}_{\alpha }^{\dagger }(j)$ and ${\gamma }_{\alpha }\mathrm{(2}j)=-\,{\rm{i}}[{c}_{\alpha }(j)-{c}_{\alpha }^{\dagger }(j)]$. They satisfy the anti-commutation relations,

13 $${\{{\gamma }_{\alpha }(m),{\gamma }_{\beta }(n)\}}_{+}=2{\delta }_{\alpha \beta }{\delta }_{mn},$$

and allow us to express the transformation in a convenient form:

14a $${\gamma }_{\alpha }\mathrm{(2}j-\mathrm{1)}={\eta }_{\alpha }[\prod _{k=1}^{j-1}{\sigma }_{\alpha }^{x}(k)]{\sigma }_{\alpha }^{z}(j)$$

14b $${\gamma }_{\alpha }(2j)={\eta }_{\alpha }[\prod _{k=1}^{j-1}{\sigma }_{\alpha }^{x}(k)]{\sigma }_{\alpha }^{y}(j)$$

14c $$\Rightarrow {\sigma }_{\alpha }^{x}(j)={\rm{i}}\,{\gamma }_{\alpha }\mathrm{(2}j-\mathrm{1)}\,{\gamma }_{\alpha }\mathrm{(2}j\mathrm{).}$$

The Klein factors *η*_*α*_ are those defined in equations (3).

The transformation relates the topological (nontopological) phase in the fermionic chains to the ferromagnetic (paramagnetic) phase of the spin system (for more details see Methods). Now we can simply translate into the spin system the unitary operator produced by, e.g., counter-clockwise braiding of Majorana modes *γ*_A_, *γ*_B_^15^:

15 $$U=\exp (\frac{\pi }{4}{\gamma }_{{\rm{A}}}{\gamma }_{{\rm{B}}})$$

(how this can be implemented may depend on the tree structure and the initial positions of *γ*_*A*_ and *γ*_*B*_). In the case of two Majorana modes that are provided by one topological segment located in a single chain before and after the braiding, the Klein factors cancel in the spin representation, so the additional spin mediating the coupling at the junction does not influence the result of the operation and is left unaffected at the end^14^.

Braiding neighbouring Majorana modes from two topological segments in different chains corresponds to a more complicated operation in the spin system. By choosing, e.g., ${\gamma }_{{\rm{A}}}={\gamma }_{1}\mathrm{(2}m-\mathrm{1)}$ and ${\gamma }_{{\rm{B}}}={\gamma }_{3}\mathrm{(2}n-\mathrm{1)}$, we obtain:

16 $$\begin{array}{rcl}{U}_{\mathrm{1,3}} & = & \exp \,[\frac{\pi }{4}\cdot {S}_{0}^{x}\prod _{j\mathrm{=1}}^{m-1}\,{\sigma }_{1}^{x}(j)\cdot {\sigma }_{1}^{z}(m)\cdot {S}_{0}^{z}\prod _{k\mathrm{=1}}^{n-1}\,{\sigma }_{3}^{x}(k)\cdot {\sigma }_{3}^{z}(n)]\\  & = & \exp \,[-{\rm{i}}\frac{\pi }{4}{S}_{0}^{y}\cdot {\sigma }_{1}^{z}(m)\prod _{j\mathrm{=1}}^{m-1}\,{\sigma }_{1}^{x}(j)\prod _{k\mathrm{=1}}^{n-1}\,{\sigma }_{3}^{x}(k)\cdot {\sigma }_{3}^{z}(n)]\\  &  & \mathop{\longrightarrow }\limits^{{\rm{effectively}}}\,\exp \,[-{\rm{i}}\frac{\pi }{4}{S}_{0}^{y}\,{\sigma }_{1}^{z}(m)\,{\sigma }_{3}^{z}(n)],\end{array}$$

if the spins outside the ferromagnetic intervals are polarised in the *x*-direction. When expressed in terms of the Pauli matrices *τ* for the two ‘topological’ qubits involved (two ferromagnetic intervals, cf. ref.^14^), this gives

17 $$U\mathrm{(1,}\,\mathrm{3)}=\exp \,[-{\rm{i}}\frac{\pi }{4}{S}_{0}^{y}\,{\tau }_{1}^{z}\,{\tau }_{2}^{z}].$$

A detailed description of this operation in the spin language is given in the section Methods.

However, for two ‘topological’ intervals in the same chain we obtain a similar expression, but without the intermediate ancillary spin:

18 $$U=\exp \,[-{\rm{i}}\frac{\pi }{4}{\tau }_{1}^{y}{\tau }_{2}^{z}].$$

Thus one obtains a two-qubit operation.

In a more general situation with arbitrary initial position of two distant braided boundaries (and associated Majorana modes), the braiding operation involves, apart from these two qubits, the ancillary spins at all intermediate vertices as well as the parity (qubit-flip) operators ∏*σ*_*x*_ for all intermediate qubit intervals. Thus, the braiding implements not a two-qubit operation but a multi-qubit operation (and also entangles qubits with the ancillas).

Here a few comments are in order: First, to achieve direct two-qubit gates between distant ‘topological’ qubit intervals, one can complement the described braiding operation with further operations involving intermediate qubits. However, for the purposes of quantum computation one does not necessarily need two-qubit logic gates between distant qubits since two-qubit gates between neighbours are sufficient, as they form a universal set of gates together with single-qubit operations. Furthermore, one can also view this subtlety from a different perspective. Instead of thinking in terms of the qubit description, one can describe the operations in terms of the fermionic (Majorana) modes involved. Then the braiding operations implement two-fermion gates, and one deals with *fermionic quantum computation*. This viewpoint may be useful for simulations of fermionic Hamiltonians (see e.g. ref.^18^), including many-body solid-state models and complex individual molecules.

A further remark concerns the symmetry and the braiding procedure: Each of the chains considered belongs to the BDI symmetry class^23^, with a time-reversal-type symmetry ${\mathscr{T}}$ such that ${{\mathscr{T}}}^{2}=1$. In a single chain (19b), a Majorana zero mode appears at each boundary between topological and non-topological regions. A vertex connecting three chains can be viewed as an edge of a 1-D system. Here the symmetry becomes crucial^24,25^. If the ${\mathscr{T}}$ symmetry is preserved by the chain coupling at the vertex, the edge (vertex) carries an integer (${\mathbb{Z}}$) ‘topological’ charge. In our case this allows for configurations with more than one Majorana zero mode at the vertex and an unwanted extra degeneracy when during the braiding procedure this vertex connects two or three ‘topological’ regions. A ${\mathscr{T}}$-breaking chain coupling, however, places the system to the D class with a ${{\mathbb{Z}}}_{2}$ invariant, and typically one (or no) Majorana zero mode exists at the vertex (cf. ref.^15^). In this case, no extra degeneracies arise during the braiding operations. In particular, this is the case for the coupling considered in ref.^14^.

## Discussion

The Jordan–Wigner transformation maps free fermionic Hamiltonians to local spin Hamiltonians. Furthermore, a nearest-neighbour hopping term is mapped to a local quadratic spin term. Some generalizations of the Jordan–Wigner transformation to higher-dimensional lattices were proposed^3–6^, which, however, map a local hopping fermionic term to a spin term involving many, often infinitely many, spins on distant sites. Other approaches (e.g. ref.^7^) use ancillary degrees of freedom but also map free fermionic terms to fourth- or higher-order spin terms.

On one hand, this implies that only some unusual spin models may be analysed with the help of such transformations. On the other hand, one could use a spin–fermion mapping to implement a fermionic model in a system built of spins (or qubits). With the motivation to implement fermionic (Majorana) degrees of freedom in a realistic qubit system, we have extended an earlier result of ref.^9^ to arbitrary tree structures. The resulting transformation maps nearest-neighbour fermionic terms to nearest-neighbour spin terms. Thus, it allows for an implementation of the Majorana physics in tree structures built out of qubit chains, extending the results of ref.^14^. This transformation provides, e.g., a spin equivalent of Majorana braiding operations. We have further shown that this construction can be generalized to arbitrary tree structures.

It must be noted that these mappings involve an enlargement of the original Hilbert space, due to the addition of spins ${\tilde{S}}_{\alpha }$ to the system. Thus, the degeneracy of all states is multiplied by a factor of 2 to the power of the number of inner vertices, but the accuracy of the mapping is not affected.

Finally, we would like to mention that experimental realizations of the 3-spin interactions crucial for our mapping were discussed in the literature (see, e.g., refs^14,26^).

## Methods

### Spin representation of two-interval Majorana braiding

**Figure 2:**
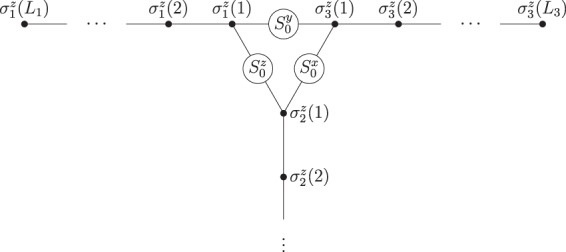
Ising spin chains in a *T*-geometry. A fermionic *T*-junction suitable for Majorana braiding^15^ has a spin representation of this structure^14^, which is described by the Hamiltonian in eq. (19). The couplings between three Ising spin chains are mediated by the components of an additional spin *S*_0_, cf. ref.^9^ and eqs (4, 5). The system can be manipulated by tuning transverse fields (not depicted here) that act on the individual spins *σ*_*α*_(*j*) of the three chains.

To obtain a spin representation for a fermionic *T*-junction of Kitaev chains, we use the transformation given in eq. (14). This yields^14^:

19a $$H=\sum _{\alpha \mathrm{=1}}^{3}\,{H}_{\mathrm{0,}\alpha }+{H}_{{\rm{int}}}^{S}$$

19b $${H}_{\mathrm{0,}\alpha }=-\,\sum _{j\mathrm{=1}}^{{L}_{\alpha }}\,{h}_{\alpha }(j){\sigma }_{\alpha }^{x}(j)-J\,\sum _{j=1}^{{L}_{\alpha }-1}\,{\sigma }_{\alpha }^{z}(j){\sigma }_{\alpha }^{z}(j+\mathrm{1)}$$

19c $${H}_{{\rm{int}}}^{S}=-\,\frac{1}{2}\sum _{\alpha \beta \gamma }|{\varepsilon }^{\alpha \beta \gamma }|\,{J}_{\alpha \beta }{S}_{0}^{\gamma }{\sigma }_{\alpha }^{z}\mathrm{(1)}{\sigma }_{\beta }^{z}\mathrm{(1)},$$

a system of Ising spin chains with a local transverse magnetic field *h*_*α*_(*j*), which corresponds to the locally tunable chemical potential in the fermionic system. Assuming *J* > 0, any interval of spins with $h\ll J$ in one of the chains is ferromagnetic, whereas $h\gg J$ results in a trivial (paramagnetic) phase. The three chains are linked by the components of an additional central spin *S*_0_ via 3-spin couplings of strength ${J}_{\alpha \beta }={J}_{\beta \alpha }$. This structure is depicted in Fig. 2.

Now we consider the spin equivalent of braiding Majorana modes from two different topological intervals in the fermionic system. The topological intervals and adiabatic shifts of their boundaries within a chain can be translated to the ferromagnetic intervals in the spin representation exactly as for a single topological interval^14^: The fermionic-parity ground states $\mathrm{|0}\rangle ,\mathrm{|1}\rangle$ of a topological interval correspond to linear combinations

20a $$|0\rangle \equiv \frac{|\uparrow \,\uparrow \,\uparrow \rangle +|\downarrow \,\downarrow \,\downarrow \rangle }{\sqrt{2}},$$

20b $$|1\rangle \equiv \frac{|\uparrow \,\uparrow \,\uparrow \rangle -|\downarrow \,\downarrow \,\downarrow \rangle }{\sqrt{2}}$$

of the ferromagnetic spin eigenstates. However, two-interval braiding cannot be effected in such a way that at most one of the 3-spin couplings in equation (19c) is relevant at each step. Therefore, the *coupler* spin *S*_0_ undergoes a generally non-trivial rotation in the process, which we will examine in the following.

Initially, the topological/ferromagnetic intervals have to be prepared, e.g., in the first and third chain at some distance from the coupler spin *S*_0_. We assume that ${J}_{12}={J}_{13}={J}_{23} > 0$ for illustration. First, we consider an initial state with the spins in both intervals and the coupler aligned in the +*z*-direction:

21 $$|{\psi }_{0}\rangle ={|\uparrow \uparrow \uparrow \odot \rangle }_{1}\otimes {|\odot \odot \rangle }_{2}\otimes {|\odot \uparrow \uparrow \uparrow \rangle }_{3}\otimes |{S}_{0}\uparrow \rangle .$$

Here the indices denote the three spin chains; the corresponding arrows indicate the spin orientation in ferromagnetic (↑/↓) and paramagnetic areas ($\odot$). They symbolize the locations of the ferromagnetic intervals in the *T*-junction geometry (Fig. 2), but the calculation does not depend on the specific interval lengths and distances to the coupler. The first step comprises shifting the right boundary of the first interval (i.e., one Majorana mode in the fermionic system) into the second chain, which results in the state

22 $$|{\psi }_{1}\rangle ={|\odot \uparrow \uparrow \uparrow \rangle }_{1}\otimes {|\uparrow \uparrow \rangle }_{2}\otimes \,{|\odot \uparrow \uparrow \uparrow \rangle }_{3}\otimes |{S}_{0}\,\uparrow \,\rangle .$$

With $|{S}_{0}\,\downarrow \,\rangle$ as initial coupler state, the spins in the second chain in eq. (22) would just be flipped compared to those in the first chain.

The non-trivial part begins when the second ferromagnetic interval is also shifted to the junction, while the spin orientation of the ferromagnetic intervals remains fixed. One can verify that for any initial state, including superpositions, the final state at this stage is always the same as in the case when the second ferromagnetic interval is adiabatically shifted towards the junction at ${J}_{13}={J}_{23}=0$, and only then these couplings are slowly turned on. This observation simplifies the further calculation. Indeed, at the end of this stage, the coupler spin rotates to adjust to the change of its effective magnetic field from the *z*-direction to the space diagonal $$\frac{1}{\sqrt{3}}\,\,(\begin{array}{c}1\\ 1\\ 1\end{array})$$:

23 $$|{\psi }_{2}\rangle ={|\odot \uparrow \uparrow \uparrow \rangle }_{1}\otimes {|\uparrow \uparrow \rangle }_{2}\otimes {|\uparrow \uparrow \uparrow \odot \rangle }_{3}\otimes [\cos \,\frac{\phi }{2}|{S}_{0}\uparrow \rangle +{e}^{{\rm{i}}\tfrac{\pi }{4}}\,\sin \,\frac{\phi }{2}\,|{S}_{0}\downarrow \rangle ]\,,$$

where $0 < \phi  < \pi \mathrm{/2}$ and $\cos \,\phi =\mathrm{1/}\sqrt{3}$. Similarly, at the next stage, when the ferromagnetic interval in the first chain is shifted away from the junction, the coupler spin adjusts to the *x*-direction:

24 $$|{\psi }_{3}\rangle ={|\uparrow \uparrow \uparrow \odot \rangle }_{1}\otimes {|\uparrow \uparrow \rangle }_{2}\otimes {|\uparrow \uparrow \uparrow \odot \rangle }_{3}\otimes \frac{1}{\sqrt{2}}[|{S}_{0}\,\uparrow \rangle +|{S}_{0}\,\downarrow \rangle ].$$

Retracting the remaining ferromagnetic interval back to the third chain is a trivial step again:

25 $$|{\psi }_{4}\rangle ={|\uparrow \uparrow \uparrow \odot \rangle }_{1}\otimes {|\odot \odot \rangle }_{2}\otimes {|\odot \uparrow \uparrow \uparrow \rangle }_{3}\otimes \frac{1}{\sqrt{2}}[|{S}_{0}\uparrow \rangle +|{S}_{0}\,\downarrow \rangle ].$$

Unlike in the case of single-interval braiding^14^, the coupler spin does not return to its initial state at the end of the operation (see, however, discussion near eq. (18)). In eqs (24), (25), we have dropped the overall phase factor that can be linked to the geometric phase of the spin evolution. It turns out to be the same for all states of interest to us (cf. eq. (26) below) and will be omitted.

For other initial conditions, the operation can be treated similarly, giving the complete result

26a $${|{\rm{\blacksquare}}{\rm{\blacksquare}}{\rm{\blacksquare}}\odot \rangle }_{1}\otimes {|\odot {\rm{\blacksquare}}{\rm{\blacksquare}}{\rm{\blacksquare}}\rangle }_{3}\otimes |{S}_{0}\,\uparrow \rangle \to {|{\rm{\blacksquare}}{\rm{\blacksquare}}{\rm{\blacksquare}}\odot \rangle }_{1}\otimes {|\odot {\rm{\blacksquare}}{\rm{\blacksquare}}{\rm{\blacksquare}}\rangle }_{3}\otimes \frac{1}{\sqrt{2}}[|{S}_{0}\,\uparrow \rangle +|{S}_{0}\,\downarrow \rangle ]$$

26b $${|{\rm{\blacksquare}}{\rm{\blacksquare}}{\rm{\blacksquare}}\odot \rangle }_{1}\otimes {|\odot \lozenge \lozenge \lozenge \rangle }_{3}\otimes |{S}_{0}\,\uparrow \rangle \to {|{\rm{\blacksquare}}{\rm{\blacksquare}}{\rm{\blacksquare}}\odot \rangle }_{1}\otimes {|\odot \lozenge \lozenge \lozenge \rangle }_{3}\otimes \frac{1}{\sqrt{2}}[|{S}_{0}\,\uparrow \rangle -|{S}_{0}\,\downarrow \rangle ]$$

26c $${|{\rm{\Delta }}{\rm{\Delta }}{\rm{\Delta }}\odot \rangle }_{1}\otimes {|\odot {\rm{\Delta }}{\rm{\Delta }}{\rm{\Delta }}\rangle }_{3}\otimes |{S}_{0}\,\downarrow \,\rangle \to -\,{|{\rm{\Delta }}{\rm{\Delta }}{\rm{\Delta }}\odot \rangle }_{1}\otimes {|\odot {\rm{\Delta }}{\rm{\Delta }}{\rm{\Delta }}\rangle }_{3}\otimes \frac{1}{\sqrt{2}}[|{S}_{0}\,\uparrow \,\rangle -|{S}_{0}\,\downarrow \,\rangle ]$$

26d $${|{\rm{\Delta }}{\rm{\Delta }}{\rm{\Delta }}\odot \rangle }_{1}\otimes {|\odot \lozenge \lozenge \lozenge \rangle }_{3}\otimes |{S}_{0}\,\downarrow \,\rangle \to {|{\rm{\Delta }}{\rm{\Delta }}{\rm{\Delta }}\odot \rangle }_{1}\otimes {|\odot \lozenge \lozenge \lozenge \rangle }_{3}\otimes \frac{1}{\sqrt{2}}[|{S}_{0}\,\uparrow \,\rangle +|{S}_{0}\,\downarrow \,\rangle ]$$

with placeholders {${\rm{\blacksquare}}$, $\lozenge$} = {↑, ↓}. The initial as well as final state of the second chain is always $|\,\odot \,\odot \,{\rangle }_{2}$. Using the parity eigenstates (20), we can verify that eqs (26) indeed correspond to the Majorana braiding. For instance,

27 $$\begin{array}{rcl}{|0\rangle }_{1}\otimes {|0\rangle }_{3}\otimes |{S}_{0}\,\uparrow \rangle  & = & \tfrac{{|\uparrow \uparrow \uparrow \rangle }_{1}+{|\downarrow \downarrow \downarrow \rangle }_{1}}{\sqrt{2}}\otimes \tfrac{{|\uparrow \uparrow \uparrow \rangle }_{3}+{|\downarrow \downarrow \downarrow \rangle }_{3}}{\sqrt{2}}\otimes |{S}_{0}\,\uparrow \rangle \\  &  & \to \,\tfrac{{|\uparrow \uparrow \uparrow \rangle }_{1}\otimes {|\uparrow \uparrow \uparrow \rangle }_{3}+{|\downarrow \downarrow \downarrow \rangle }_{1}\otimes {|\downarrow \downarrow \downarrow \rangle }_{3}}{2}\otimes \tfrac{|{S}_{0}\uparrow \rangle +|{S}_{0}\downarrow \rangle }{\sqrt{2}}\\  &  & +\,\tfrac{{|\uparrow \uparrow \uparrow \rangle }_{1}\otimes {|\downarrow \downarrow \downarrow \rangle }_{3}+{|\downarrow \downarrow \downarrow \rangle }_{1}\otimes {|\uparrow \uparrow \uparrow \rangle }_{3}}{2}\otimes \tfrac{|{S}_{0}\uparrow \rangle -|{S}_{0}\downarrow \rangle }{\sqrt{2}}\\  & = & \tfrac{1}{\sqrt{2}}{[|0\rangle }_{1}\otimes |0{\rangle }_{3}\otimes |{S}_{0}\uparrow \rangle +|1{\rangle }_{1}\otimes |1{\rangle }_{3}\otimes |{S}_{0}\,\downarrow \rangle ].\end{array}$$

In addition to the ferromagnetic intervals, the superposition involves the coupler spin, in accordance with the expressions (16), (17), which are compatible with the results (26).

Note that the coupling *J*_13_ is not necessary for the operation we considered here. The choice of *J*_13_ = 0 simplifies the coupler rotations and leads to the same results.
